# Conductance interaction identification by means of Boltzmann distribution and mutual information analysis in conductance-based neuron models

**DOI:** 10.1186/1471-2202-13-S1-P100

**Published:** 2012-07-16

**Authors:** Roberto Santana, Concha Bielza, Pedro Larrañaga

**Affiliations:** 1 Department of Computer Science and Artificial Intelligence, University of the Basque Country, Spain; 2 Departamento de Inteligencia Artificial, Universidad Politécnica de Madrid, Spain

## 

Recent research has focused on the causal paths that explain how neuron ionic-conductances interact to produce a particular electrophysiological behavior. Pharmacological blockage methods, neuromodulators and genetic knockouts are among the techniques used to study this issue. In this paper we propose a probabilistic approach based on the computation of the Boltzmann distribution and the mutual information of conductance interactions to learn higher-order, not necessarily pair-wise, potential co-regulation mechanisms from a database of the crustacean stomatogastric ganglion pyloric circuit models. The original database was built from experimental data obtained from lobster stomatogastric neurons [2,3]. The eight currents in the single-compartment model are based on the lobster stomatogastric ganglion neurons currents.

The basic idea of our work is to assign a probability value *p*(*x*) to each neuron model depending on whether or not it satisfies a given electrophysiological property. To assign these values we use the Boltzmann probability distribution, commonly used in statistical physics to associate a probability with a system state according to its energy [4]. From the Boltzmann distribution we compute the mutual information to measure the strength of interaction between a pair of conductances at the time of producing a particular electrical activity.

The particular characteristics of silent neurons were captured in the uneven distribution of bivariate marginal probabilities computed from the Boltzmann distribution. Among all conductance pairs, the highest bivariate probabilities are reached for conductance pairs (g_KCa_,g_Kd_), (g_Na_,g_KCa_) and (g_CaT_,g_CaS_). With respect to previous analysis of the group of silent neurons, the correlation (g_CaT_,g_CaS_) was the only significant linear correlation identified for silent models in [1]. There were another three conductance relationships reported in [1] that fitted statistical criteria for correlations, but did not appear to have a linear relationship. They were (g_H_,g_leak_), (g_KCa_,g_Kd_) and (g_KCa_,g_CaT_). All these correlations were identified as significant correlations of the computed Boltzmann distribution

Our results show that probabilistic modeling based on the Boltzmann distribution can capture potential co-regulations that are not captured by the correlation analysis. The extension to capture higher-order dependencies between conductances is also straightforward. Furthermore, our results indicate that mutual information analysis allows a more detailed visualization of the structure of the conductance landscape for conductance-based neuron models.
